# OrthoMaM: A database of orthologous genomic markers for placental mammal phylogenetics

**DOI:** 10.1186/1471-2148-7-241

**Published:** 2007-11-30

**Authors:** Vincent Ranwez, Frédéric Delsuc, Sylvie Ranwez, Khalid Belkhir, Marie-Ka Tilak, Emmanuel JP Douzery

**Affiliations:** 1 Université Montpellier 2, CC064, Place Eugène Bataillon, 34 095 Montpellier Cedex 05, France; 2 CNRS, Institut des Sciences de l'Evolution (UMR 5554), CC064, Place Eugène Bataillon, 34 095 Montpellier Cedex 05, France; 3 Centre de Recherche LGI2P, Ecole des Mines d'Alès, Site EERIE Parc scientifique G. Besse, 30035 Nîmes Cedex 1, France

## Abstract

**Background:**

Molecular sequence data have become the standard in modern day phylogenetics. In particular, several long-standing questions of mammalian evolutionary history have been recently resolved thanks to the use of molecular characters. Yet, most studies have focused on only a handful of standard markers. The availability of an ever increasing number of whole genome sequences is a golden mine for modern systematics. Genomic data now provide the opportunity to select new markers that are potentially relevant for further resolving branches of the mammalian phylogenetic tree at various taxonomic levels.

**Description:**

The EnsEMBL database was used to determine a set of orthologous genes from 12 available complete mammalian genomes. As targets for possible amplification and sequencing in additional taxa, more than 3,000 exons of length > 400 bp have been selected, among which 118, 368, 608, and 674 are respectively retrieved for 12, 11, 10, and 9 species. A bioinformatic pipeline has been developed to provide evolutionary descriptors for these candidate markers in order to assess their potential phylogenetic utility. The resulting OrthoMaM (Orthologous Mammalian Markers) database can be queried and alignments can be downloaded through a dedicated web interface .

**Conclusion:**

The importance of marker choice in phylogenetic studies has long been stressed. Our database centered on complete genome information now makes possible to select promising markers to a given phylogenetic question or a systematic framework by querying a number of evolutionary descriptors. The usefulness of the database is illustrated with two biological examples. First, two potentially useful markers were identified for rodent systematics based on relevant evolutionary parameters and sequenced in additional species. Second, a complete, gapless 94 kb supermatrix of 118 orthologous exons was assembled for 12 mammals. Phylogenetic analyses using probabilistic methods unambiguously supported the new placental phylogeny by retrieving the monophyly of Glires, Euarchontoglires, Laurasiatheria, and Boreoeutheria. Muroid rodents thus do not represent a basal placental lineage as it was mistakenly reasserted in some recent phylogenomic analyses based on fewer taxa. We expect the OrthoMaM database to be useful for further resolving the phylogenetic tree of placental mammals and for better understanding the evolutionary dynamics of their genomes, i.e., the forces that shaped coding sequences in terms of selective constraints.

## Background

Mammalian systematics has been a pioneering field in the use of molecular sequence data for inferring phylogenetic relationships. Molecular phylogenies at different levels of the mammalian evolutionary tree have accumulated since the seminal studies published in the early 1990s. Among the first genes to be used was the mitochondrial Cytochrome b (*MT-CYB*) [1] which since became the "barcode" marker for mammals with more than 20,000 sequences currently available representing about 2,000 species. The mitochondrial 12S rRNA gene has also early been considered but its use was limited by its less straightforward alignment [2]. Acknowledging the limits of single gene phylogenies, the potential of complete mitochondrial genomes for reconstructing placental orders relationships was early explored, but with a somewhat limited success as judged *a posteriori *[3]. In parallel, the first efforts to identify single-copy orthologous nuclear markers for mammalian phylogenetics have been made from conserved, large-sized exons. The exon 1 of the Retinol Binding Protein 3 (*RBP3*) – also known as the Interphotoreceptor Retinoid-Binding Protein (*IRBP*) – was the first to be developed [4] later followed by the exon 28 of the von Willebrand Factor gene (*VWF*) [5]. Since then, other nuclear genes have acquired the status of "standard" mammalian phylogenetic markers. This is for example the case of the intronless Recombination Activating Gene 1 (*RAG1*) and α-2B Adrenergic Receptor (*ADRA2B*) genes, the Growth Hormone Receptor (*GHR*) exon 10, the c-myc proto-oncogen (*MYC*) exon 3, and the Breast Cancer Associated protein 1 (*BRCA1*) exon 11. Either used in single gene phylogenies at their beginnings or in combination later [6,7], this handful of markers has proven to be useful for unravelling unsuspected clades at different levels of the mammalian taxonomy, like for instance Afrotheria [8], Cetacea + Hippopotamidae [9], and the grouping of shrews and hedgehogs to the exclusion of moles within Eulipotyphla [10].

The choice of these useful markers has nevertheless been mainly empirical. In fact, their initial development has been almost entirely dependent upon the availability in public databases of human, murine, bovine, and canine sequences for allowing primer design. This historical constraint in marker choice involved that the phylogenetic informativeness of these genes is likely to be non optimal for many of the phylogenetic studies in which they have been used [11]. Selecting the genes with the appropriate resolving power for a given phylogenetic problem is a difficult task, and theoretical work has so far provided only limited insight for guiding this choice [12,13]. In practice, however, it has long been realized that there is an optimal evolutionary rate associated with a given phylogenetic question [14], and empirical procedures such as saturation plots [15] have been designed to evaluate the limits in resolving power of a given molecular marker.

In mammals, the first attempt at specifically selecting multiple nuclear genes for tackling a circumscribed phylogenetic question was made by Murphy and co-workers who specifically targeted genes scattered throughout the mammalian genome to resolve the earliest placental divergences [16]. Their pragmatic approach was based on the initial selection of exons long enough for easy PCR amplification from whole genomic DNA (> 200 bp) and for which the nucleotide identity between human and mouse ranged between 80 and 95%. This simple procedure was successful at identifying a dozen of new phylogenetically informative nuclear markers for resolving the long-standing question of the evolutionary relationships among placental orders [16].

Mammalian phylogenetics is now turning into phylogenomics [17] with such large-scale sequencing initiatives as the ENCODE project [18,19] and the NISC Comparative Vertebrate Sequencing Program [20]. The availability of mammalian whole genome sequences provides a gold mine for the identification of new phylogenetic markers to further resolve the mammalian tree at different taxonomic levels. However, in this new genomic era, the main problem perhaps resides in the determination of orthology relationships among the different genomes. Bioinformatic tools have been developed for processing whole genome sequences such as INPARANOID [21] and OrthoMCL [22] resulting in dedicated databases of clusters of orthologous groups for eukaryotes [23,24]. A recent comparison of different orthology detection strategies has shown that phylogenetically based methods perform better than classical similarity search based methods [25]. Accordingly, the 2007 version of the EnsEMBL database now implements such a phylogenetically-based strategy using maximum likelihood and tree reconciliation methods for orthology assignment among vertebrate genomes [26].

In an effort to synthesize all these genomic information, we built upon the EnsEMBL database for constructing a mammalian centred database called OrthoMaM (Orthologous Mammalian Markers). Our aim is to provide a flexible resource for identifying new candidate markers for future use in mammalian phylogenetic studies. Similar approaches based on available genomic data have been recently conducted in plants [27] and ray-finned fishes [28] but they include their own determination of orthology in the corresponding bioinformatic pipelines. By directly relying on the EnsEMBL orthology assessment procedure, our approach has the advantage of being relatively easy to update as more mammalian complete genomes will become available and annotated.

We focused on orthologous exons rather than on full-length transcripts in order to provide biologists with single continuous fragments potentially amplifiable from genomic DNA. Working with RNA extraction followed by RT-PCR would require a quality of tissue preservation that is not achieved in the vast majority of cases. Moreover, working with genomic DNA avoids the practical problems induced by potential differences of intron length among taxa during the PCR amplification, provided that exons are specifically targeted. We selected individual exons of more than 400 bp long. Increasing this arbitrary threshold might preclude the use of old tissue samples or museum specimens that often contain altered total DNA. Also, lowering this threshold length would involve keeping a total of 7,206 human, murine, and canine exons among which the shorter is only 84 bp long. The minimum length for an exon to be included in the database was thus set up to 400 bp because it offers a reasonable compromise between technical (PCR) constraints, the number of selected candidates, and subsequent sequencing efforts.

Until now, the choice of phylogenetic markers for mammalian systematics has been governed more by historical constraints than by explicit criteria. This is the reason why we developed a bioinformatics pipeline to derive evolutionary descriptors related to the potential phylogenetic informativeness of each exon. Quantifying the substitution pattern of genes is important to understand the potential biases that might affect phylogenetic inferences [29]. Ideally, a good marker would have an optimal evolutionary rate for the given phylogenetic question, equilibrated and homogeneous base frequencies [30,31], and homogeneous distribution of site variability [11]. Yet the characteristics of a valuable marker depends on the goals of the study it will be used for, and certainly also vary from one investigator to another. Therefore, rather than subjectively selecting a subset of these candidate exons, we provide evolutionary descriptors for all of them. The values of our evolutionary descriptors are indicated for each exon on individual web pages with links to EnsEMBL for full description of the loci. The corresponding sequence alignment and its associated maximum likelihood phylogenetic tree with model parameter estimates are also presented. Note that this phylogeny should be considered cautiously since it might not be optimal in terms of topology because of the use of a suboptimal model (see below), but it should nevertheless provide reliable estimates of model parameters [32]. In any case, markers cannot be selected directly from the ML topology they have produced in order to avoid any potential misuse of the database biased by *a priori *phylogenetic beliefs.

A number of these evolutionary characteristics can be queried directly through the web-interface. The value and reliability of some of these descriptors are strongly dependent with one another. For example, the global GC content is strongly related to the GC percentage at the third codon position. Moreover, the variance on model parameter estimates will be reduced with longer sequences. The OrthoMaM web-interface allows the user to take these interdependencies into account. On the one hand, one can impose sequences longer than 1000 bp when expecting precise estimates of model parameters. On the other hand, the sequence length may not really matter if the goal is to build a supermatrix with reduced GC bias by combining markers displaying roughly equilibrated base frequencies. Combined queries allow the easy retrieval of orthologous genes that are present in a given number of species for automating phylogenomic supermatrices assembly.

## Construction and content

### Using the EnsEMBL orthology information

The gene annotation available through the EnsEMBL database includes orthology information [26]. This annotation is based on phylogenetic analyses of clusters of homologous sequences corresponding to the longest transcript of each gene. Two homologous sequences can be annotated as being paralogue or orthologue depending on their relative position in the corresponding phylogeny. In this phylogeny, when two sequences from different taxa are closer to each other than to any other sequence of the corresponding taxa, they are said to be 1:1 orthologues. Such an orthology assessment is particularly interesting as it avoids markers having similar copies in the genome that can interfere during the amplification process. The quality of the EnsEMBL annotation is ensured by the analysis of a plethora of phylogenies of homologous sequences [26]. This annotation requires computation facilities far beyond those available in most laboratories, but allows predicting orthology and paralogy relationships much more accurately than the classical reciprocal best hits approach [25]. We therefore exploited this precious annotation for further analyses rather than trying to compete with it.

The procedure used for detecting candidate markers for placental phylogenetics from whole genomes involves the following steps. First, we focused on human (*Homo sapiens*), mouse (*Mus musculus*) and dog (*Canis familiaris*), whose genomes have been fully sequenced with a high coverage, are well annotated, and are evolutionary divergent enough so that a gene shared by these three taxa is likely to be conserved among placental mammals. We selected genes that are predicted by EnsEMBL to be 1:1 orthologues between *Homo*:*Mus*, *Homo*:*Canis *and *Mus*:*Canis*. Second, for each such gene, we retrieved the longest transcript available for the *Homo sapiens *gene and for all its 1:1 orthologues among the 12 studied taxa (the latter three, plus *Pan troglodytes*, *Macaca mulatta*, *Rattus norvegicus*, *Oryctolagus cuniculus*, *Bos taurus*, *Dasypus novemcinctus*, *Loxodonta africana*, *Echinops telfairi*, and *Monodelphis domestica*). Then, we considered each exon of the human transcripts and searched for the corresponding orthologous exon in transcripts of other species. We assumed that the most similar exon of the orthologous transcript is actually the orthologous exon provided its sequence shared more than 50% of similarity with the human sequence. This similarity test just checks for the fact that there is an exon that matches with the one currently tested. More difficult problems about gene orthology, gene annotation, or pseudogene occurrence are already handled upstream by the EnsEMBL annotation system [33].

**Figure 1:**
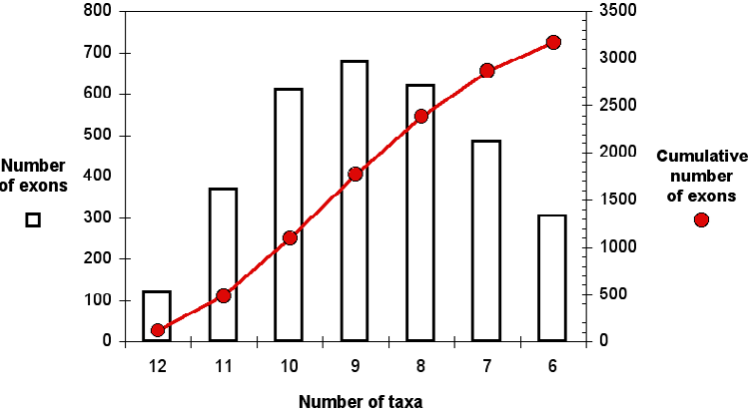
**Relation between the number of candidate exons and the number of mammalian taxa**. Raw and cumulative numbers of exons as a function of the number of taxa for which they are retrieved are respectively given by the histogram and the red circles.

All orthologous exons that matched the previous criteria are stored in our database as a set of candidate phylogenetic markers. A total of 3,170 exons among 2,498 genes has been identified, among which the majority is available for 9 taxa (Figure 1). A subset of 118 exons is available for all 12 mammals, and, for fair comparisons among markers, will subsequently constitute the core dataset to illustrate the OrthoMaM properties. This figure is quite low as compared to the 3,170 initial markers available, but is easily explained by the low coverage of recently sequenced genomes. For example, 1,252 candidate markers were found for the 2X-coverage genome of *Dasypus novemcinctus*, versus 2,623 for the *Bos taurus *genome sequenced with a more comprehensive 6X-coverage.

### A bioinformatic pipeline to describe the evolutionary dynamics of exons

The following section describes the tools used by our pipeline to estimate the descriptors of the molecular evolutionary properties of the markers. A suite of phylogenetic analyses was conducted to characterize each exonic marker. Since these analyses are time consuming, we relied on a Beowulf-class cluster supercomputer. Dedicated scripts have been developed to parallelize this step so that the database can be regularly updated. This was necessary due to the frequent updates of the EnsEMBL database. Moreover, our scripts are flexible enough to easily integrate new species when they become available as well as phylogenetic software updates. This ensures the upgradeability of our OrthoMaM database.

First, the DNA sequences of exons were aligned with the help of amino acid translation using the transAlign software [34]. Because some exon sequences were shorter than others at 5'- or 3'-extremities (for example because of either lower genomic coverage or too preliminary annotation), alignment extremities were trimmed for sites with missing nucleotides for at least half of the taxa. For a preliminary, fast screening of potential misaligned exons or divergent paralogues, a neighbor-joining (NJ, [35]) analysis on uncorrected pairwise distances was conducted with PAUP* version 4b10 [36]. Alignments yielding a NJ tree with a total branch length (TBL) exceeding 2 substitutions per site were discarded.

Second, base composition of the exons was characterized. For this purpose, we used the relative composition variability (RCV) [31] whose exact formula is:

$$RCV=\frac{\sum_{i=1}^{t}\left(|A_{i}-A^{*}\right|+|C_{i}-C^{*}|+|G_{i}-G^{*}|+|T_{i}-T^{*}|)}{s.t^{2}}$$

where *A*_*i*_, *C*_*i*_, *G*_*i*_, *T*_*i *_denote the numbers of each nucleotide for taxon *i*, and A*, C*, G* and T* are averages across the *t *taxa, and *s *is the total number of sites. RCV therefore quantifies the extent of overall base composition variability among sequences.

**Figure 2:**
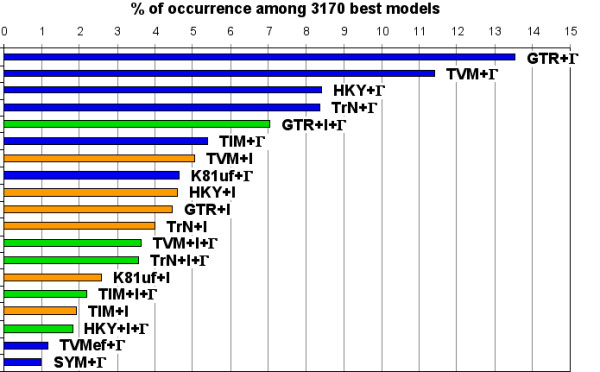
**A variety of models account for the substitution patterns of the 3170 exons**. Best-fitting models of sequence evolution selected for 99% of the candidate markers are here illustrated. The 22 other best-fitting models that describe the remaining 1% of the exons are here not represented. Blue, orange, and green colours respectively depict models involving Gamma distribution (Γ), invariable sites (I), and a combination thereof (Γ+I). Abbreviations: *GTR *(General Time Reversible), *TVM *(Transversional model), *HKY *(Hasegawa, Kishino, Yano 85), *TrN *(Tamura-Nei 93), *TIM *(Transitional model), *K81 *(Kimura 81), and *SYM *(Symmetrical model); *ef *(equal base frequencies), and *uf *(unequal base frequencies).

Third, we used maximum likelihood (ML) analyses [37] to describe and compare the nucleotide substitution pattern among exons. The best fitting model for each of the 3,170 candidate markers was chosen by ModelTest version 3.7 [38] based on the Akaike Information Criterion [39]. Among the 3,170 best models identified, we report those that occurred the most frequently (Figure 2). Nineteen different substitution models contribute for best fitting 99% of the exon alignments, and they involve either a Γ distribution (8 times/19), or a fraction of invariable sites (6/19), or a combination thereof (5/19). The best-fitting model that is most often selected by ModelTest is GTR+Γ, i.e., the General Time Reversible (GTR) model of nucleotide substitution [40] with substitution rate heterogeneity among sites described by a Gamma distribution of shape α [41]. For this reason, and for fair comparison of model parameters among exonic alignments, OrthoMaM reports GTR+Γ estimates for all candidate markers. Specifically, we used PAUP* to provide users with ML estimates of α parameter, base frequencies, and A-C, A-G, A-T, C-G, and C-T GTR substitution rates between nucleotides (relative to G-T = 1.0).

Fourth, an important descriptor of the utility of a phylogenetic marker is its relative evolutionary rate: faster (respectively slower) evolving markers will be more suitable for lower (respectively deeper) taxonomic levels. In a first approximation, the TBL of the highest-likelihood tree is a reasonable descriptor of the evolutionary rate of a given exon. However, the TBL will preclude fair comparisons among different exons when the taxon sampling differs: the higher the species number, the longer the TBL. To circumvent this problem, we used the Super Distance Matrix (SDM) approach [42], with a three-step procedure: (i) The ML tree inferred from each of the 3170 exons was converted into a matrix of additive distances by computing the path-length between each pair of species. (ii) Each of the 3170 matrices was brought closer to the others by a factor (*α*_*p*_), according to the least-squares criterion; this operation is equivalent to multiplying by *α*_*p *_every branch length of the initial trees. (iii) Optimal values of the *α*_*p *_parameters are calculated following SDM* in reference [42], and, as *α*_*p *_are inversely proportional to the evolutionary rates, 1/*α*_*p *_values provide a measure of rate heterogeneities among exons even if the number of taxa differs. In addition, the quality of the highest-likelihood tree was also measured through its treeness, i.e., the relative contribution of the sum of internal branches to the TBL [31]. If a star tree is inferred from a given marker, this lack of phylogenetic signal will be reflected by a treeness value of zero. Conversely, the higher the treeness, the better the resolution of the internal parts of the tree will be.

In order to better evaluate the contrast level of evolutionary dynamics among codon positions, some calculations were also conducted separately on first, second, and third codon positions. The distribution of variability over the three codon positions was evaluated for each exonic marker by calculating the contribution of first, second, and third codon positions relative to the total number of variable sites. This allows distinguishing between exons with variability concentrated on third positions versus exons with a more even distribution over the three codon positions. Such a distinction might be useful for maximizing the number of phylogenetically informative characters when selecting an exon for further sequencing in a given taxonomic group. For example, the widely used exon 11 of *BRCA1 *has been shown to have an almost equal distribution in the number of substitutions among the three codon positions [43,44]. This property associated with a relatively slow overall substitution rate made *BRCA1 *a particularly informative marker for resolving placental mammal earliest divergences [44,45].

## Utility and discussion

### User interface

**Figure 3:**
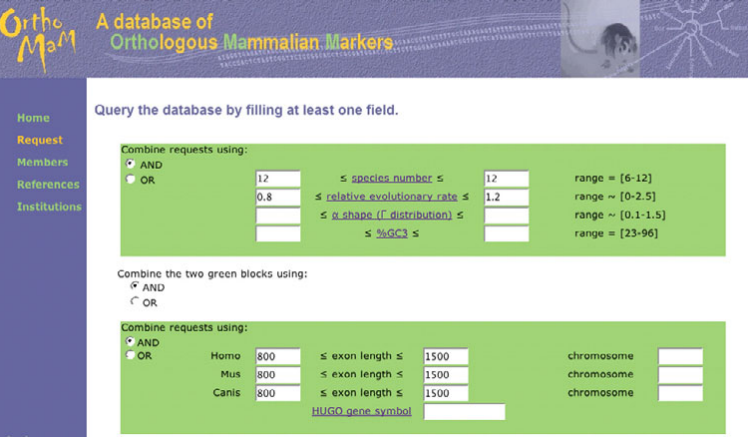
**Screenshot of the query form of OrthoMaM**. In this example, the user requests orthologous exons available for all 12 mammals, characterized by a relative evolutionary rate ranging from 0.8 to 1.2, and a length ranging from 800 to 1,500 bp for human, mouse, and dog.

**Figure 4:**
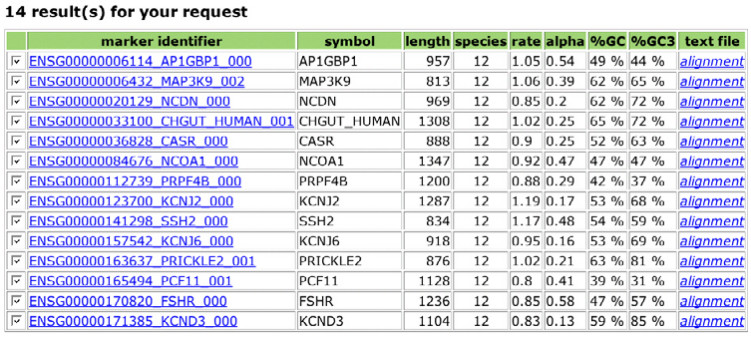
**Screenshot of the result sheet**. The result of the query previously submitted (see Figure 3) is shown. Fourteen candidate exons are recovered with a recapitulation of their phylogenetic descriptors.

**Figure 5:**
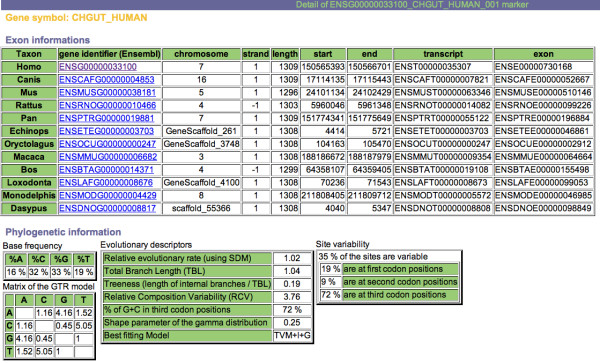
**Screenshot of the recapitulation of the evolutionary descriptors of *CHGUT_HUMAN***. Details about the available exon of each mammal are presented, including EnsEMBL gene/transcript/exon identifiers, chromosome location, and chromosome coordinates. Phylogenetic information is also provided, including base frequencies, GTR matrix of the relative substitution rates between nucleotides, various evolutionary descriptors, and a summary of site variability. The alignment and the corresponding highest-likelihood tree are also given (not shown here).

To search the OrthoMaM database, a request form is available (Figure 3). A range of values of the evolutionary dynamics descriptors can be given, either independently or in combination. This includes the number of species for which the orthologous exons are available (set to a maximum of 12 for the current version), the relative evolutionary rate of the markers (as measured by the SDM procedure on highest-likelihood trees), their level of among-site substitution rate heterogeneity as measured by the α shape of the Γ distribution, and their percent of GC at third codon positions. Moreover, this query can be associated with a query about genomic descriptors including gene symbol, human-murine-canine chromosome numbers, and exon length for the three reference species *Homo*, *Mus *and *Canis*. The result of the query is visualized as a recapitulative table of the different descriptors with link to the alignment in Fasta format (Figure 4). Once the marker is chosen by the user, the synopsis of all its descriptors can also be obtained with direct links to EnsEMBL for details on each individual sequence (Figure 5).

### Evolutionary descriptors

To illustrate the importance of providing various descriptors of the evolutionary dynamics of the 3,170 exonic markers currently included in the OrthoMaM database, we summarized the range of values for some of them (Table 1). For fair comparison among markers with respect to missing taxa, only descriptor values for the 118 exons present in all 12 species are here presented. People interested in getting the maximum number of molecular characters from a single PCR amplification will focus on longer exons. For example, the alignment of the longest conserved exon (EnsEMBL gene ENSG00000189079: AT rich interactive domain 2 gene [*ARID2*], human exon 15) spans ~2.8 kb among the 12 mammals. On average, exonic alignments contain one-third (35.7%) of variable sites, among which two-third (68.4%) occur at third codon positions. Some exons retain > 90% of their variability at third codon positions (the maximum is 93.4%). Others possess a more evenly distributed variability, with a maximum variability of 34.4% on first codon positions, and 28.6% at second codon positions. Evenly distributed site variability is associated with a high value of the α-shape parameter and potentially reduces the level of homoplasy as nucleotide substitutions could accumulate at each codon position over the whole exon [44]. About base composition, the mean GC3 is 58.7%, with a very wide range from 29.3% to 89.3%. Nucleotide substitution patterns are fairly variable as well, with e.g. the relative C-T substitution rate ranging from 1.1 to 34.5, and the average transition/transversion rate ratio ranging from 0.8 to 6.5. Moreover, the relative evolutionary rate among exons, as estimated by SDM, exhibited a more than 10-fold variation between slowest- and fastest-evolving candidates.

**Table 1:** Descriptors of the evolutionary dynamics of the 118 exons available for 12 mammals (human, chimp, macaque, mouse, rat, rabbit, cow, dog, elephant, tenrec, armadillo, and opossum).

**DESCRIPTORS**	**MEAN**	**SE**	**MIN**	**MAX**
**Exon length**	800	465	405	2883
**Variability**	35.7	10.0	15.7	60.6
**% var 1**^st^	19.5	6.2	5.9	34.4
**% var 2**^nd^	12.1	7.1	0	28.6
**% var 3**^rd^	68.4	12.8	42.0	93.4
**α (Γ distribution)**	0.4	0.2	0.1	1.3
**%GC**	52.8	7.5	39.5	69.4
**%GC3**	58.7	15.2	29.3	89.3
**RCV (× 1000)**	6.8	2.9	1.7	16.3
**A-C**	1.5	0.9	0.4	6.7
**A-G**	6.1	3.4	1.4	24.4
**A-T**	1.0	0.7	0.2	3.8
**C-G**	1.1	0.7	0.1	3.6
**C-T**	7.7	4.8	1.1	34.5
**G-T**	1.0	0	1.0	1.0
**Ti/Tv**	2.8	0.9	0.8	6.5
**TBL**	0.9	0.4	0.2	1.9
**Relative rate (SDM)**	1.0	0.4	0.2	2.6

Mean, standard-error (SE), minimum (MIN) and maximum (MAX) values are given for exon length, variability (% of variable sites on the complete alignment), relative variability (% var) among codon positions (% of the variable sites that respectively occurred on first [1st], second [2nd], and third [3rd] codon positions), substitution rate heterogeneity among sites (α), Guanosine + Cytosine content on all (GC) and third (GC3) codon positions, relative composition variability (RCV), relative GTR substitution rates (A-C to G-T), average transition/transversion rate ratio (Ti/Tv), total branch length (TBL) of the tree, and relative evolutionary rate (as measured by the SDM procedure).

### Development of new phylogenetic markers

We illustrate the potential utility of the OrthoMaM database with the development of two new markers for placental phylogenetics. We focused on the 118 candidates retrieved for all 12 mammals, and we chose among exons with a length ranging between 800–1500 bp for the three-species core (human, mouse and dog), with an intermediate relative rate of evolution, i.e., SDM value ranging from to 0.8 to 1.2 (Figure 3). Fourteen candidate exons satisfied the combination of these three criteria, among which *CHGUT_HUMAN *(1,308 bp) and *NCOA1 *(1,347 bp) were the longest (Figure 4). We thus selected the corresponding alignments containing sequences that are orthologous to exon 4 of the human CHondroitin sulfate GlucUronylTransferase gene (EnsEMBL gene reference ENSG00000033100), and to exon 11 of the human Nuclear receptor CO-Activator 1 gene (ENSG00000084676).

Our *in silico *approach for development of new markers was then validated by the successful amplification and sequencing of *CHGUT_HUMAN *exon 4 orthologues for species belonging to two of the most evolutionary distant groups of placental mammals: xenarthrans (member of Atlantogenata, the clade of southern origin), and rodents (member of Boreoeutheria, the clade of northern origin) [46]. The xenarthran species was the anteater *Tamandua tetradactyla*. The rodent species were a caviomorph (the degu, *Octodon degu*), a sciurid (the Guianan squirrel, *Sciurus aestuans*), a dipodid (the lesser Egyptian jerboa, *Jaculus jaculus*), and two sigmodontine muroids (the MacConnell's rice rat, *Oryzomys macconnelli*, and the Guiana bristly mouse, *Neacomys guianae*). Similarly, we obtained sequences of *NCOA1 *exon 11 orthologues for rodents, including *Octodon*, *Jaculus*, *Oryzomys*, *Neacomys*, and also a glirid (the garden dormouse, *Eliomys quercinus*) and an anomalurid (a scaly-tailed flying squirrel, *Anomalurus *sp.).

All ethanol preserved tissues were extracted using QIAamp DNAminikit following manufacturer (*QIAGEN*) instructions. A 1.1 kb portion of *CHGUT_HUMAN *exon 4 orthologues was amplified by Polymerase Chain Reaction (PCR) with forward 1F (5'-GCYCAGATCCGGAACCTGAC-3') and reverse 1R (5'-AACCGGAGGAAAACATCCATCACC-3') primers. A 1.2 kb portion of *NCOA1 *exon 11 orthologues was amplified with forward 1F (5'-CAGTGGCCTTTCTCCTCAAG-3') and reverse 1R (5'-ACCTTTACRTCATCCAGGC-3') primers. Amplification reactions were carried out in 20 μl including 50 μM of each primer, dNTP (200 μM), 1× Taq buffer, 1 U Taq polymerase (*Triple Master PCR System Eppendorf*) and 50–100 ng genomic DNA. Amplifications were performed in Mastercycler gradient (*Eppendorf*) using denaturation at 94°C (4 min), followed by 29 temperature cycles of 94°C (20 sec), 53°C to 60°C (20 sec) and 72°C (1 min 30 sec), with a final extension at 72°C (10 min). The temperature gradient was necessary for PCR optimisation on all taxa. PCR products were purified from 1% agarose gels with the DNA gel extraction kit (*Millipore*) and directly sequenced using the 1F/1R external primers, and two internal ones: 3F (5'-GTGGARATCCTGCCYATGCC-3') and 2R (5-CACCTGGGAMGGKGCCTC-3') for *CHGUT_HUMAN*, and 2F (5'-CAAACAATTCATTTCCTCC-3') and 2R (5'-GCATGCCGTAACTGCTG-3') for *NCOA1*. The Bigdye Terminator kit v1.1 (*Applied Biosystem*) was used and sequencing reactions were run on an ABI 310 (*Applied Biosystem*) automated sequencer. Sequences have been deposited in the EMBL database under accession numbers AM900835 to AM900846.

**Figure 6:**
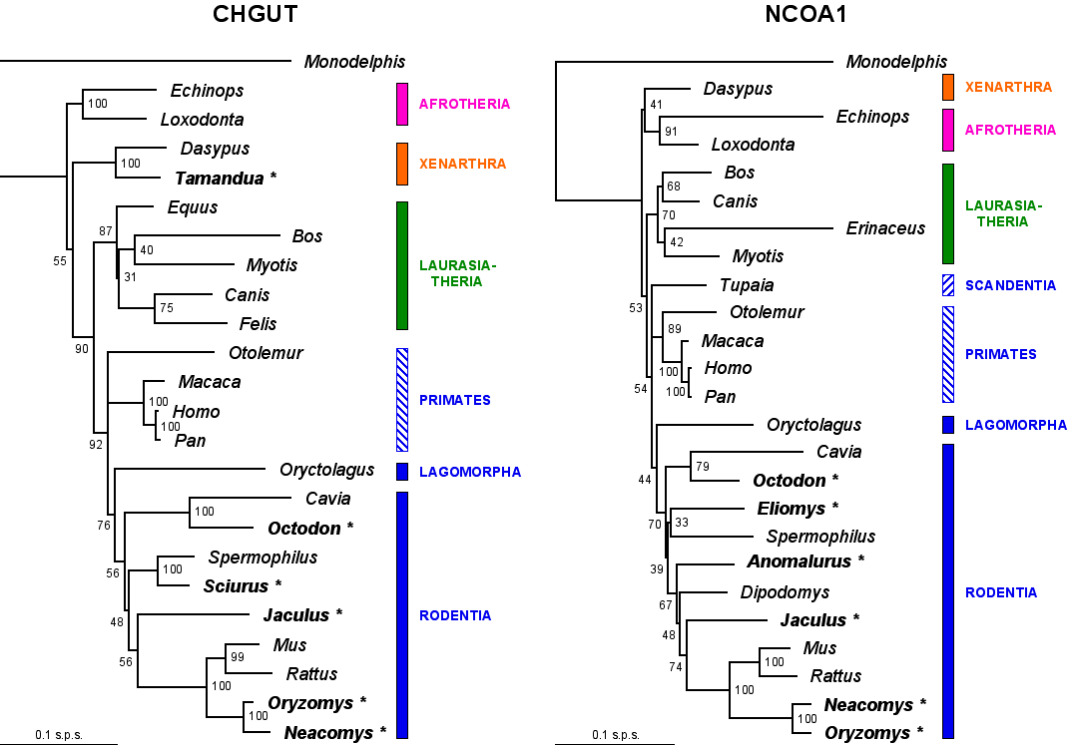
**Maximum likelihood phylogenies reconstructed from alignments of orthologues to the human CHondroitin sulfate GlucUronylTransferase (*CHGUT_HUMAN*) exon 4 [left], and to the human Nuclear receptor CO-Activator 1 gene (*NCOA1*) exon 11 [right]**. Values on nodes are ML bootstrap support. Taxa for which the *CHGUT_HUMAN *and *NCOA1 *exons were obtained in vivo are indicated in bold + asterisk. Sequences from other taxa were recovered *in silico *from traces, pre-assembled, and assembled EnsEMBL genomes. The name of major clades is provided on the right, and blue rectangles correspond to Euarchontoglires mammals. The outgroup *Monodelphis *is drawn with midpoint rooting. Horizontal branch lengths are proportional to the DNA divergence (same scale for both exons = 0.1 nucleotide substitutions per site). Maximum likelihood details about *CHGUT_HUMAN*|*NCOA1 *phylograms are respectively as follows: log-likelihoods are ln*L *= -8,868.8|8,899.4, and estimates of model parameters are: %A = 15.6|29.2, %C = 32.6|27.5, %G = 33.0|21.2, and %T = 18.8|22.1 for base frequencies ; A-C = 1.11|0.63, A-G = 4.61|4.04, A-T = 1.38|0.68, C-G = 0.44|0.54, C-T = 4.17|3.31, and G-T = 1.00 for GTR relative substitution rates ; and α = 0.30|0.51 for rate heterogeneity among sites.

Newly obtained *CHGUT_HUMAN *and *NCOA1 *sequences were added to the ones of the 12 mammals included in the OrthoMaM database, complemented by other species downloaded from ongoing genome projects and traces (*Equus caballus*, *Myotis lucifugus*, *Felis catus*, *Tupaia belangeri*, *Otolemur garnettii*, *Cavia porcellus*, *Spermophilus tridecemlineatus*, and *Dipodomys ordii*). Maximum likelihood analyses of the final alignments under the best-fitting GTR+Γ model yielded trees conforming to the current view of the placental phylogeny (Figure 6). The four major clades Afrotheria, Xenarthra, Laurasiatheria, and Euarchontoglires are recovered. Among the latter group, the rabbit (Lagomorpha) is the sister group of rodents, forming the Glires clade. Monophyletic rodents subdivide into three subclades respectively containing Guinea-pig related, squirrel-related, and mouse-related species, in agreement with recent multigene phylogenies of Rodentia [47,48].

### A phylogenomic approach on placental mammals

**Figure 7:**
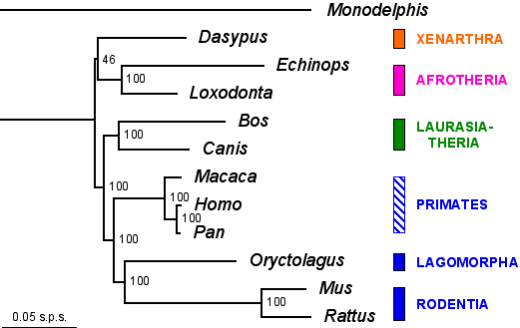
**Maximum likelihood phylogeny reconstructed from a 94-kb alignment of 118 concatenated orthologous exons present for all 12 mammals**. The log-likelihood of the phylogram is ln*L *= -407,623.4, and estimates of model parameters are: %A = 25.9, %C = 27.0, %G = 25.0, and %T = 22.1 for base frequencies ; A-C = 1.29, A-G = 4.69, A-T = 0.72, C-G = 1.21, C-T = 5.75, and G-T = 1.00 for GTR relative substitution rates ; INV = 31.7% for the fraction of invariable sites, and α = 0.74 for rate heterogeneity among the remaining sites. Values on nodes are ML bootstrap support. Sequences from the 12 taxa were *in silico *recovered from assembled EnsEMBL genomes. The outgroup *Monodelphis *is drawn with midpoint rooting. Horizontal branch lengths are proportional to the DNA divergence (scale = 0.05 nucleotide substitutions per site).

As a second illustration of the utility of the OrthoMaM database, we used it in a phylogenomic perspective. To that aim, we combined the 118 exons present for all 12 species (human, chimp, macaque, mouse, rat, rabbit, cow, dog, elephant, tenrec, armadillo, and opossum), and obtained a supermatrix of 94,739 sites. We only used exons detected in these 12 mammals by our automatic, bioinformatic pipeline, to minimize the amount of missing character states which is as low as 4.8% due to incomplete 5'/3' coverage and gapped sites. The highest-likelihood topology calculated by PAUP* was well-resolved, as evaluated through ML bootstrap support, except for the position of the placental root (Figure 7). Rodents (*Mus *and *Rattus*) and the lagomorph (*Oryctolagus*) are grouped into Glires as a sister-group of catarrhine primates (*Homo*, *Pan *and *Macaca*) to form Euarchontoglires. Euarchontoglires branch with Laurasiatheria (here represented by *Bos *and *Canis*) into Boreoeutheria, all with 100% bootstrap support.

Our topology is thus fully compatible with the new understanding of placental mammal phylogeny revealed by early multigene analyses to the exception of the position of the root [6,16,49]. However, other recent studies based on phylogenomic data sets claimed support for a closer evolutionary relationship between primates and carnivores relative to muroid rodents [50-52]. Such results appear all the more surprising given that the monophyly of rodents plus primates relative to carnivores and cetartiodactyls observed in Figure 7 is also supported by a large body of independent evidences including multigene mitochondrial and nuclear DNA phylogenies [7,44,53-55], indel protein signatures [56], and SINEs insertions [57].

The main commonality among these three recent phylogenomic studies is their use of reduced taxon samplings associated with whole genome sequences, a situation where statistical phylogenetic inconsistency is particularly prone to occur [29,58]. Interestingly, in our dataset, the evolutionary rate of muroid rodents appears 2.7 times faster than the one of primates as attested by relative branch lengths of the ML phylogram (Figure 7). Nevertheless, *Mus*, *Rattus *and *Oryctolagus *grouped with *Macaca*, *Homo *and *Pan*, whereas a long-branch attraction (LBA) phenomenon [59] would have attracted muroids towards the distant marsupial outgroup.

In order to test for this LBA hypothesis, we restricted our analysis to the 8-taxon sampling of Cannarozzi et al. [50] including only human, chimp, macaque, mouse, rat, cow, dog, and opossum. The use of a sub-optimal and under-parameterized model, i.e. GTR without Γ+INV, led to a ML topology conforming to the results obtained by Cannarozzi et al. [50]: *Mus *+ *Rattus *branched to the most basal position among placentals, with a 99% bootstrap support for grouping *Bos *+ *Canis *with primates. However, under the better-fitting GTR+Γ+INV model, the ML analysis recovered with 100% bootstrap support the initial topology (*cf*. Figure 7) in which primates and rodents are grouped together to the exclusion of carnivores + cetartiodactyls. Here, the more sophisticated model seems to correct the long-branch attraction artefact of muroid rodents towards the opossum branch.

Further investigations were conducted by adding the rabbit in order to break the long isolated muroid branch. Under the GTR model (without Γ+INV), Glires appeared monophyletic and branched with primates to the exclusion of *Canis *and *Bos*, and the ML bootstrap support for the euarchontoglires clade was 77%. A denser taxon sampling (i.e., the addition of *Oryctolagus*) therefore reduces the LBA phenomenon, and here compensates for model underparameterization. The use of the better-fitting GTR+Γ+INV model confirmed this trend, and provided 100% bootstrap support for grouping rodents with primates. Finally, the reanalysis of the 12-taxon OrthoMaM supermatrix of 118 exons under a GTR model but without Γ+INV yielded 100% bootstrap within boreoeutherians for the topology of Figure 7. These analyses confirm the crucial impact of taxon sampling for accurate phylogenetic inference, especially when long isolated branches are involved [58,60]. Moreover, accounting for among-sites rate variation through a Gamma distribution is important to accurately discriminate among alternative topologies, especially when the taxon sampling is depauperate [61,62].

From our ML analyses, it seems that the basal position of rodents observed in the three recent phylogenomic studies [50-52] is likely a LBA artefact associated with the use of a reduced taxon sampling and/or inadequate phylogenetic reconstruction methods. Our study strongly supports the phylogenetic affinities of rodents with primates to the exclusion of carnivores (Figure 7), and adds credit to the view that they should no longer be considered as contentious [63]. The same phylogenetic relationships among rodents, primates and carnivores is obtained from a GTR+Γ ML analysis of the supermatrix of characters resulting from the combination of all 3,170 exons of the OrthoMaM database (3,047,860 sites for 12 taxa ; 32% missing character states ; results not shown).

The only unresolved node in our phylogenomic analysis (Figure 7) involves the position of the root of the placental tree. There has been debate to know whether xenarthrans are sister-group of all remaining placentals (the Epitheria hypothesis) [57,64], or branch with Afrotheria (the Atlantogenata hypothesis; [65]), or with Boreoeutheria (the basal Afrotheria hypothesis [53,54]). Here, the combination of the 118 exons yielded bootstrap support of 45% for Atlantogenata (the highest-likelihood branching pattern), 46% for Epitheria, and 9% for basal Afrotheria. Indel signatures [46] and larger datasets [66,67] actually seem to favour the Afrotheria + Xenarthra branching. However, it has been argued that the latter two results might reflect an artefact of using concatenated likelihood models whereas partitioned models rather favour the basal Afrotheria hypothesis [68].

## Conclusion

The OrthoMaM database provides an intuitive interface for querying thousand of orthologous exons of potential use in placental mammal systematics. It also allows the easy retrieval of large sets of conserved orthologous exons among the available mammalian genomes to perform phylogenomic analyses. The evolutionary descriptors characterizing each candidate marker are of particular interest for phylogenetic marker choice and for comparative analyses of molecular evolution at the genome scale. The bioinformatic pipeline behind OrthoMaM allows envisioning that the database will be dynamically updated on a regular basis to follow the evolution of EnsEMBL and thereby ensure its accuracy. We expect the OrthoMaM database to prove useful for further resolving the phylogenetic tree of mammals and for understanding the selective pressures that shaped the evolution of their genomes.

## Availability and requirements

Project name: OrthoMaM (*Ortho*logous *Ma*mmalian *M*arkers);

Project home page: ;

Operating system(s): Platform independent;

Programming language: Java, PHP, XML, XSLT;

License: None;

Any restrictions to use by non-academics: None.

## Authors' contributions

VR and EJPD initiated the study, conceived and implemented the bioinformatic pipeline. VR, SR and KB constructed the database and designed the web interface. MKT carried out PCR amplifications and sequencing. EJPD, FD and MKT performed the phylogenetic analyses for the two case studies. VR, FD and EJPD wrote the manuscript. All authors read and approved the final manuscript.
